# Impacts of changes in intestinal flora on the metabolism of *Sprague–Dawley* rats

**DOI:** 10.1080/21655979.2021.2000242

**Published:** 2021-12-02

**Authors:** Chengfei Wang, Dong Yan, Jianrong Huang, Yongtao Li

**Affiliations:** State Key Laboratory for Diagnosis and Treatment of Infectious Diseases, the First Affiliated Hospital, College of Medicine, Zhejiang University, Hangzhou Zhejiang Province, China

**Keywords:** Intestinal flora, metabolomics, gc/ms, real-time pcr, *salmonella enteritidis*

## Abstract

Changes in intestinal flora affect the health and cause metabolic diseases of the host. The extent to which the impact of different changes in intestinal flora would have on the metabolism of an individual has not been reported. This study aims to investigate the effect of different changes in intestinal flora on the metabolism of Sprague–Dawley (SD) normal rats’ individuals. Forty-eight SD rats were randomly divided into 6 groups (8 rats per group), which were treated with normal saline, probiotics, nonpathogenic *Escherichia coli, Salmonella enteritidis*, gentamicin, and magnesium sulfate, respectively. After 7 days, the ileum of each group of rats was collected and real-time polymerase chain reaction was used to analyze the composition of intestinal flora. And gas chromatography/mass spectrometry (GC/MS) was used to analyze plasma metabolic profile. The results revealed that the decrease in alanine content in the probiotics group was statistically significant, while the alanine content in the nonpathogenic *Escherichia* group increased significantly. Alanine, leucine, isoleucine, and serine decreased significantly in the *Salmonella* group. Proline and butyric acid decreased significantly in the gentamicin group. The principal component analysis showed significant differences in the *Salmonella* group compared with other test groups. Overall, the most significant metabolic changes were observed in SD rats in the *Salmonella* group, while a great similarity was observed in the probiotics, *Escherichia* group, and gentamicin groups compared with the normal group. Changes in intestinal flora had a certain impact on the metabolism in SD rats, especially on amino acid levels.

## Introduction

Intestinal flora is a complex micro-ecological system in the human body. Under normal circumstances, the intestinal flora maintains a dynamic equilibrium and interacts with the host, which can be seen as a bacterial organ in the body, with multiple functions [1,2]. Because intestinal flora can express glycoside hydrolase and polysaccharide lyase that do not exist in the human body, its main metabolic functions are fermentation and decomposition of food residues and mucus secreted by intestinal epithelial cells. Meanwhile, it can perform anaerobic metabolism for proteins and peptides [3–5]. Previous studies have demonstrated that compared with peer children of normal weight, intestinal *Bifidobacterium* decreased in obese children while *enterococci* increased. Furthermore, a high-fat diet can effectively change the dominant bacteria in the intestinal tract, resulting in glycogen deposition in the liver, increase in the body fat, and weight gain [6,7]. Therefore, changes in intestinal flora affect the host health and the process of metabolic diseases, such as obesity and diabetes [8–10]. Thus, maintaining intestinal flora balance is an important factor affecting human health [11]. It is closely related to human metabolism.

However, in the course of many diseases, such as cirrhosis, acute pancreatitis, shock, severe burns, intestinal infections, and multiple organ failure, this fine balance of intestinal flora is often disturbed, causing changes in the components and composition of the intestinal flora [12–14]. In pressure emergency, repeated use of antibiotics, and a variety of noninfectious diarrhea, normal intestinal flora of the human body can be changed, resulting in severe clinical symptoms [15,16]. Thus, change in intestinal flora is a common clinical phenomenon. A previous study also showed that the intestinal flora would be changed in acute liver injury, manifesting as a significant decrease in *lactobacilli* and obvious increase in *Escherichia coli* [17]. It has also been reported that changes in intestinal flora can affect serum metabolites of rats before and after pregnancy, thus affecting maternal and offspring health [18]. In addition, Li et al. [19] showed a significant increase in *Escherichia coli* and *enterococci* and a significant decrease in *lactobacilli* in type 2 diabetic rats. The above studies confirm that the intestinal flora changes differently in patients with different diseases.

Metabolomics is a powerful tool that can well assess the pressure response caused by environmental changes, diseases, and toxins. With the innovation and technological improvements of derivatization methods, metabolites in complex biological samples such as urine, blood, tissues, and so on, can be derived so as to be gasified and analyzed by gas chromatography/mass spectrometry (GC/MS). The greatest advantage of GC/MS is the ability to identify metabolites with a mature NIST database. The GC/MS-based metabolomics study has become one of the important tools for metabolomics research [20]. Gavaghan et al. [21] then used high performance liquid chromatography to analyze metabolites in the urine of Sprague–Dawley (SD) normal rats and identified two different subpopulations. Scholars Robosky et al. [22] used two groups of SD rats from the same source and phenotype in their study and placed them in different environments. The rats were fed the same food. It was found that the two groups of SD rats differed in urinary marker metabolites and colonic colony structure. These studies confirm that the different phenotypes are due to different intestinal flora. However, differences in the effects of changes in gut flora on individual metabolites have not yet been reported. Therefore, the aim of this experimental study was to investigate the extent to which changes in intestinal flora affect the metabolism of the body. Different gavages were used in this study to simulate clinical changes in common intestinal flora, including increase in probiotics, *Enterobacteria* proliferation, infectious diarrhea, use of antibiotic, and noninfectious diarrhea. Real-time polymerase chain reaction (PCR) was used to detect several dominant bacteria in the healthy body to evaluate the changes in the flora in each experimental group. The correlation between the changes in intestinal flora and those in metabolic substances in the experimental groups were analyzed and compared, in order to reveal the different impact of the changes in intestinal flora on the body.

## Materials and methods

### Experimental design

Specific pathogen-free (SPF) Sprague–Dawley (SD) male rats (Zhejiang Academy of Medical Sciences, Hangzhou, China) were selected. They weighed 190–230 g and were fed in a nonspecific pathogen environment (SPF laminar flow tract) with the temperature of 22 ± 2°C. The rats had a free access to water and standard rat chow, and were exposed to 12/12-h bright and dark cycle on a daily basis. All animals were under humanitarian care. The experimental program was approved by the Animal Care Committee of Zhejiang University and consistent with relevant state regulations.

The 48 SD rats were randomly divided into 6 groups with 8 rats in each group: (I) the normal group received 2 ml of physiologic saline by daily gavage; (II) the probiotics group received 2 mL live *Lactobacillus fermentum* solution [2.0 × 10^10^ colony-forming unit (CFU)/mL] by daily gavage; (III) the *Escherichia* group received with 2 mL living nonpathogenic *E. coli* 25,922 solution (2.0 × 10^10^ CFU/ mL) by daily gavage; (IV) the *Salmonella* group received 2 mL living *S. enteritidis* solution (2.0 × 1010 CFU/mL) by daily gavage; (V) the gentamicin group received 2 mL gentamicin (40,000 units) by daily gavage; and (VI) the magnesium sulfate group received 2 mL magnesium sulfate (10%) by daily gavage. After 7 days of continuous gavage, the rats were injected intraperitoneally with 50 mg/kg ketamine hydrochloride, and inhalation of ether was used for anesthesia. Inferior vena cava was extracted, and the serum was isolated to perform metabolomics analysis of the rats. Then, contents of the terminal ileum were used to analyze intestinal flora. All gavage bacteria were stored in our laboratory.

## Steps of serum derivatization

A total of 200 μL serum was added to 800 μL of methanol (protein was removed and enzyme activity was inhibited). The mixture was vortexed for 1 min; then, 20 μL ribose alcohol mother liquor (0.2 mg/mL, pure water configuration, internal reference) was added to the mixture and vortexed for 1 min, followed by heating at 70°C for 10 min and centrifuging at 10,000 rpm for 10 min. The supernatant was extracted to be added to 500 μL pure water and 250 μL chloroform, which was centrifuged at 4000 rpm for 15 min (the aforementioned steps were carried out in a 1.5-mL cryopreservation tube, and the following steps were carried out in a dry and closable glass tube). The supernatant was dried with nitrogen (60°C). Then, 50 μL methionamine solution (20 mg/mL, pyridine) was added to the dried supernatant and vortexed for 1 min. The solution was heated at 70°C for 60 min and vortexed for 1 min. Subsequently, 99 μL methyl-trimethyl-silyl-trifluoroacetamide (MSTFA) and 1 μL templated mesoporous carbons (TMCs) were added to the solution. Finally, the solution was placed at room temperature for 2 h and then the sample was loaded to be examined with gas chromatography–mass spectrometry (GC/MS). This experiment was performed with the serum of each group of rats.

## GC/MS parameter setting

This experiment used an Agilent gas system 6890 N, and capillaries with the specification of ZB-5 MS (Phenomenex, USA) were used for sample injection. A total of 2.0 μL sample was injected each time with the temperature at the injection inlet of 270°C and the total flow rate of 504 mL/min. The experiment used the non-diversion mode with helium as a carrier gas. The maximum column temperature was 300°C. The temperature was first maintained at 70°C for 2 min, then increased at the rate of 15°C/min, followed by increasing to 280°C to be maintained for 9 min. The scanning range was 60–800 m/z. The peak signals and corresponding peak values were identified by the AMDS software (National Institute of Standards and Technology, Gaithersburg, MD) along with the NIST v1.0.0.12 mass spectrometry database.

## Detection of intestinal flora

A specimen of the intestinal terminal content was taken. The 16s DNA V3 segment of intestinal microflora was amplified by 16s DNA universal primer real-time–PCR (primer sequence: 338 F-G: 5ʹ-CGCCCGCCGCGCGCGGCGGGCGGGGCGGGGGCACGGGGGGCCTACGGGAGGCAGCAG-3ʹ, 518 R: 5ʹ-ATTACCGCGGCTGCTGG-3ʹ) [23]. DNA solution was extracted according to the DNA extraction kit (purchased from QIAGEN, USA). The extracted DNA was quantitatively tested using the fluorescence quantitative PCR (fluorescence quantitative PCR was purchased from BIO-RAD, USA) and synthetic specific primers (Table 1) for five common intestinal floras, including Bacteroides, Bifidobacterium, Lactobacillus, Enterococcus, and Enterobacteriaceae, so as to identify the composition of the intestinal flora in different experimental groups. The reaction was carried out using a 20-μL reaction system, and the reaction conditions were as follows: pre-degeneration at 95°C for 3 min; then, 40 cycles of common PCR amplification were performed (95°C for 30 s, 56°C for 40 s, and 72°C for 30 s). The intensity of fluorescence was measured for 10 s after the amplification of each cycle to avoid interference of the primer dimer, secondary structure, and so on. Finally, extension at 72°C was performed for 5 min. The results were represented as log10 CFU/g intestinal contents [24–26]. All primers were synthesized by Shanghai Yingjun Biotechnology Co., Ltd.

**Table 1.** PCR primer information of the intestinal flora fluorescence quantification

Target group	Sequence (5ʹ–3ʹ)
Bacteroides-Prevotella group	GAAGGTCCCCCACATTG
	CAATCGGAGTTCTTCGTG
Bifidobacterium genus	GGGTGGTAATGCCGGATG
	TAAGCCATGGACTTTCACACC
Lactic acid bacteria	AGCAGTAGGGAATCTTCCA
	ATTTCACCGCTACACATG
Enterococcus faecalis	AACCTACCCATCAGAGGG
	GACGTTCAGTTACTAACG
Enterobacteriaceae	CATTGACGTTACCCGCAGAAGAAGC
	CTCTACGAGACTCAAGCTTGC

## Statistical methods

SPSS18.0 software was used for analysis. The data were represented as mean ± standard deviation (SD). Comparison of the mean value for multiple samples used one-way analysis of variance. The *P* value was <0.05, as the difference was considered to be statistically significant. Principal component analysis (PCA) was used according to the relative value of GC/MS of serum metabolites in each experimental group.

## Results

### Analysis of fecal flora in each experimental group

**Figure 1.**
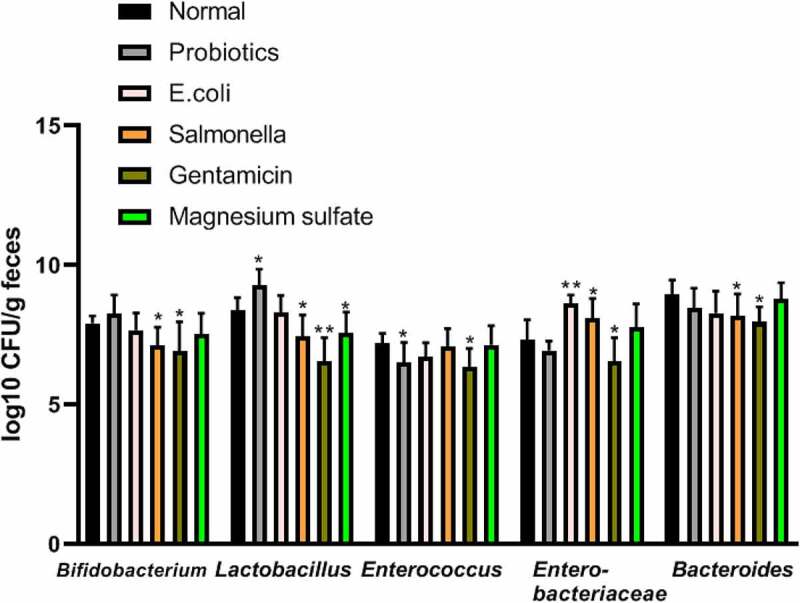
Analysis of fecal flora in terminal ileum in different experimental groups

Compared with the normal group, Lactobacillus increased significantly in the probiotics group (9.27 ± 0.59 *vs*. 8.38 ± 0.45 log10CFU/g, respectively; P < 0.05), while *Enterococcus* significantly decreased and *Enterobacteria* increased obviously in the *Escherichia* group (8.64 ± 0.29 *vs*. 7.32 ± 0.72 log10CFU/g, respectively; P < 0.05). *Bifidobacterium* and *Lactobacillus* significantly decreased in the Salmonella group (7.14 ± 0.64 *vs*. 7.92 ± 0.26 log10CFU/g; 7.45 ± 0.77 *vs*. 8.38 ± 0.45 log10CFU/g respectively; P < 0.05), while enterobacteria increased significantly compared to the control group (8.10 ± 0.71 *vs*. 7.32 ± 0.72 log10CFU/g, respectively; P < 0.05). The Gentamicin treatment reduced the population of both aerobic and anaerobic bacteria significantly compared to the control group. *Lactobacillus* significantly decreased in the magnesium sulfate group compared to the control group (7.57 ± 0.75 *vs*. 8.38 ± 0.45 log10CFU/g, respectively; P < 0.05) (Figure 1).

The Normal group was gavaged with normal saline; the probiotics group was gavaged with *lactobacilli* fluid; the *E.coli* group was gavaged with *E. coli* solution; the *Salmonella* group was gavaged with *S. enteritidis*; the gentamicin group was gavaged with gentamicin (40,000 units); and the magnesium sulfate group was gavaged with magnesium sulfate (10%). Compared with the normal group, *P < 0.05 and **P < 0.001. Data were represented as mean ± SD; log10 CFU/g feces.

## Analysis and identification of serum metabolites

The components of the 24 metabolites were analyzed and identified through peak area and NIST mass spectrometry database, which contained 11 kinds of amino acids, 2 kinds of monosaccharides, and 3 kinds of short-chain fatty acids (Table 2). Flying time was the peak time of metabolites.

**Table 2.** Serum metabolites identified by GC/MS analysis

	Number of flying time (min)	Components of the metabolites
1	7.4462	Propionic acid
2	8.4806	Alanine
3	10.0279	Butyric acid
4	11.5496	Valine
5	12.2591	Urea
6	13.0798	Leucine
7	13.2337	Phosphate
8	13.6525	Isoleucine
9	13.9432	Glycine
10	14.0971	Succinic acid
11	15.5161	Serine
12	16.2171	Threonine
13	18.2859	Aspartic acid
14	19.3374	Proline
15	21.7908	Phenylalanine
16	24.4409	Xylitol
17	28.1083	Mannose
18	28.1767	Lysine
19	28.416	Glucose
20	30.2198	Palmitic acid
21	31.6132	Inositol
22	33.1776	Linoleic acid
23	33.7333	Stearic acid
24	49.3946	Cholesterol

## Comparison of the relative value of GC/MS peak area of serum metabolites

**Figure 2.**
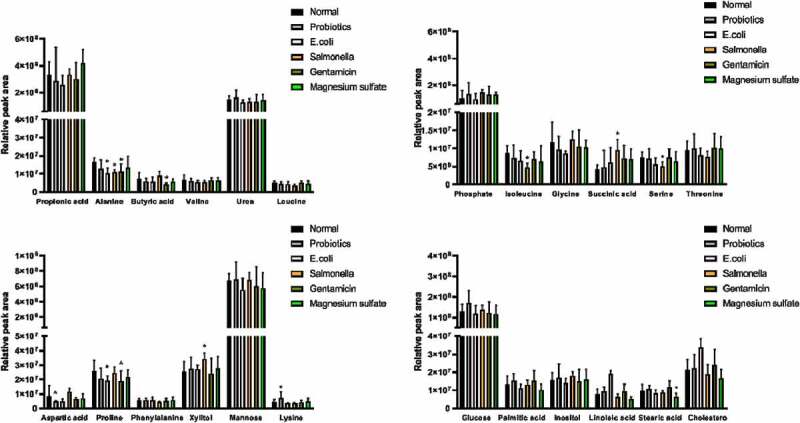
Comparison of the results of the relative value of GC/MS peak area in the serum metabolites

The decrease in Aspartic acid content in the probiotics group was statistically significant (4.65E+06 ± 6.22E+05 *vs*. 8.46E+06 ± 7.58E+06 GC/MS peak area, respectively; P < 0.05), while the alanine content in the nonpathogenic Escherichia group significantly decreased compared to the control group (1.01E+07 ± 3.21E+06 *vs*. 1.65E+07 ± 2.44E+06 GC/MS peak area, respectively; P < 0.05). A variety of amino acids (alanine, isoleucine, and serine) significantly decreased in the Salmonella group compared to the control group (GC/MS peak area, respectively; P < 0.05) but the content of Succinic acid and Xylitol significantly decreased (9.43E+06 ± 2.94E+06 *vs*. 4.28E+06 ± 1.09E+06; 3.41E+07 ± 4.36E+06 *vs*. 2.52E+07 ± 7.31E+06 GC/MS peak area, respectively; P < 0.05). Proline and butyric acid significantly decreased due to the inhibition of intestinal flora in the gentamicin group compared to the control group (Figure 2).

The Normal group was gavaged with normal saline; the probiotics group was gavaged with *lactobacilli* fluid; the E.coli group was gavaged with *E. coli* solution; the *Salmonella* group was gavaged with *S. enteritidis*; the gentamicin group was gavaged with gentamicin (40,000 units); and the magnesium sulfate group was gavaged with magnesium sulfate (10%).The peak represented the relative concentration of metabolites in each experimental group, which was expressed as mean ± SD; compared with the normal group *P < 0.05.

## Principal component analysis

**Figure 3.**
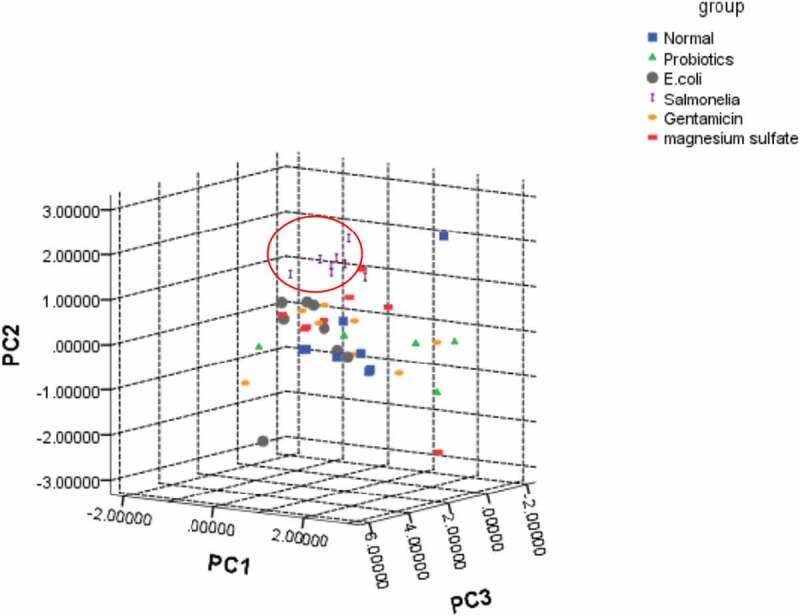
A three-dimensional scattergram of metabolites in each experimental group

SPSS software was used to perform PCA for the metabolites in each experimental group. The first three PCAs extracted 44.589% (PC1), 12.075% (PC2), and 8.658% (PC3) of the independent variables to perform canonical discriminant analysis for the clustering of each experimental group. Then a three-dimensional scattergram was plotted. The *Salmonella* group was well distinguished (Figure 3).

The Normal group was gavaged with normal saline; the probiotics group was gavaged with *lactobacilli* fluid; the E.coli group was gavaged with *E. coli* solution; the *Salmonella* group was gavaged with *S. enteritidis*; the gentamicin group was gavaged with gentamicin (40,000 units); and the magnesium sulfate group was gavaged with magnesium sulfate (10%).

## Discussion

The ecological balance of intestinal flora is an important physiological balance of the body. Any external intervention to the physiological stability of the body would cause damage to the stability of the internal environment, resulting in the changes in key metabolic process in the corresponding cells of the body [27]. Studies have shown that intestinal flora and its activities are associated with metabolic diseases such as diabetes, obesity, and so on; Changes in the maternal intestinal flora by diet can even affect the metabolic processes of body for the offspring, leading to obesity. Moreover, a negative correlation was observed between obesity and the proportion of staphylococcal/bacillus cells [28–30]. These studies suggest that different diseases lead to alterations in the metabolism of the organism’s intestinal flora. In the present study, we simulated the clinical changes of common intestinal flora by giving different gavage methods with SD rats and analyzed the terminal ideal flora of rats by Real-time PCR for comparison. The results showed that the probiotic group showed a significant increase in *Lactobacillus*, while *Enterococcus* decreased. Enterobacteriaceae increased significantly in the *Escherichia* group. *Bifidobacterium, Lactobacillus*, and *Bacteroides* in the *Salmonella* group decreased simultaneously, while Enterobacteriaceae increased. Intestinal flora showed a comprehensive reduction in the gentamicin group. A significant reduction was observed in *Lactobacillus* in the magnesium sulfate group. This suggests that dietary differences can lead to differences in the composition of the intestinal flora.

In addition, we found that changes in intestinal flora by different ways could lead to varying degrees of metabolic changes in SD rats. Compared with SD rats in the normal group, aspartic acid in the probiotic group showed a significant reduction, while lysine showed a significant increase. In the *Escherichia* group, the contents of phenylalanine increased compared with the normal group, and the contents of other amino acids decreased with a significant decrease in proline and alanine. However, various amino acids in the rat serum in the *Salmonella* group decreased to different degrees, in which the decreases of alanine, isoleucine, and serine were significant. In particular, aspartic acid content increased. In the gentamicin group, the content of leucine was slightly higher than that of the normal group, and the content of other amino acids decreased. These results suggest that such specific alterations can be corrected with changes in the intestinal flora. This is in agreement with the findings of Robosky [22] and Rohde [31]. They analyzed that the differences in the composition of intestinal flora were caused by the metabolic differences of SD rats. It has been shown that for rats with high-fat diet, Enterobacteriaceae decreased and Streptomyces increased, leading to increase in branched-chain amino acid metabolic diseases such as leucine, isoleucine, and valine. Meanwhile, glucogenic amino acids such as alanine, proline, and so on, decreased [32,33]. However, when the body had serious diseases, such as severe viral hepatitis and cirrhosis accompanied by hepatic encephalopathy, intestinal flora would have varying degrees of changes, manifesting as obvious increase in the proportions of Enterobacteriaceae and Streptococcus bacteria and significant decrease in the proportion of Rhizoctoniaceae bacteria. Moreover, the metabolic manifestations were the increase in fragrant amino acids (phenylalanine, tyrosine), and normal or slight decrease in branched-chain amino acids (valine acid, leucine, isoleucine) [2,34,35].

Propionic acid and butyric acid are two short-chain fatty acids, which are the endpoints of intestinal fermentation metabolism of carbohydrates as well as the anaerobic metabolism of proteins and peptides under the role of flora. Moreover, their relative amounts depended on the presence of specific bacteria. For healthy people, increases in the bifidobacteria and lactobacilli contribute to the catabolism of proteins in the colon [36,37]. In this experiment, the contents of propionic acid and butyric acid in the Salmonella group were lower than those in the normal group, which decreased in the probiotics and gentamicin groups. On the contrary, the propionic acid increased in the magnesium sulfate group, while butyric acid decreased. However, in previous studies, the fermentation of prebiotics (inulin-type fructans) increased the abundance of intestinal propionate and butyrate [38,39].

Previous studies demonstrated a difference in the intestinal flora between obesity and non-obesity, and bacteroid in the distal colon of obesity reduces significantly compared with non-obesity, suggesting that changes in intestinal flora affect the fat metabolic process of the body [40]. In the present study, compared with the normal group, levels of palmitic acid and linoleic acid increased in the probiotic and gentamicin groups, which decreased in the Salmonella, Escherichia, and magnesium sulfate groups. After probiotics were fed to sterile mice implanted with infant intestinal flora, a series of changes in tissue metabolism were caused, which affected the energy of the body, and fat and amino acid metabolism, suggesting that intestinal flora played its role in maintaining metabolic balance of the body. Supplement of probiotics Bifidobacterium can effectively reduce the weight gain of the human body, hepatic steatosis, and other related metabolic disorders [41,42].

An unsupervised multivariate pattern recognition PCA analysis was performed for identified metabolites so as to reveal the change regulations of metabolic profile in each experimental group. From Figure 1, the metabolic profile of the Salmonella group was gradually away from the healthy state, indicating that the impact of inflammation of the body was relative high on the metabolism. Previous studies suggested that intestinal metabolites can be involved in the control of inflammation by controlling mechanisms of neutrophil chemotaxis, and their activity may affect the regulation of inflammatory processes in enteritis [43,44].

## Conclusion

In this study, we found that changes in intestinal flora by different ways had different impacts on the metabolism of SD rats, among which Salmonella enteritidis had the greatest effect on the metabolism of the body. This study provides new ideas for understanding the pathological mechanisms of clinical diseases. However, due to the complexity of the intestinal flora itself, the mechanism of its interaction with the body had not been fully elucidated, and the exact mechanism of participating in the metabolism of the body was still unclear, which needed to be further investigated.

## Highlights

1. Changes in intestinal flora had a certain impact on the metabolism in SD rats;
2. Probiotics induced a significant decrease in alanine in SD rats;
3. Non-pathogenic *Escherichia coli* induced an increase in alanine in SD rats;
4. *Salmonella* induced a decrease in alanine, leucine, isoleucine and serine in SD rats.
